# P-2177. The Prevalence of Respiratory Viral Pathogen Detection on Nasopharyngeal Specimens Evaluated by Multiplex Polymerase Chain Reaction Among Asymptomatic Children

**DOI:** 10.1093/ofid/ofae631.2331

**Published:** 2025-01-29

**Authors:** Milissa U Jones, Emily Parsons, Heidi Adams, Jessica Wilkins, Priscilla Kobi, Justin L Robinson, Allison M Malloy

**Affiliations:** Uniformed Services University, Bethesda, Maryland; Uniformed Services University, Bethesda, Maryland; Henry Jackson Foundation, Bethesda, Maryland; USUHS, Ashburn, Virginia; HJF/IDCRP, USUHS, Bethesda, Maryland; Uniformed Services University of the Health Sciences, Gaithersburg, Maryland; Department of Pediatrics, Uniformed Services University of the Health Sciences, Bethesda, MD, USA, Bethesda, Maryland

## Abstract

**Background:**

While current viral detection methods demonstrate high sensitivity, their clinical relevance remains inexact. We evaluated the prevalence of respiratory viral pathogen detection (RVPD) using multiplex polymerase chain reaction (M-PCR) and its correlation with nasal immune cell quantities and reported respiratory viral symptoms (RVS) in asymptomatic or pauci-symptomatic children.

Demographic and Clinical Characteristics of 20 Children at Enrollment in the Pediatric Respiratory Co-infection and Immunologic Response Study (Peds RECON)

**Figure:**
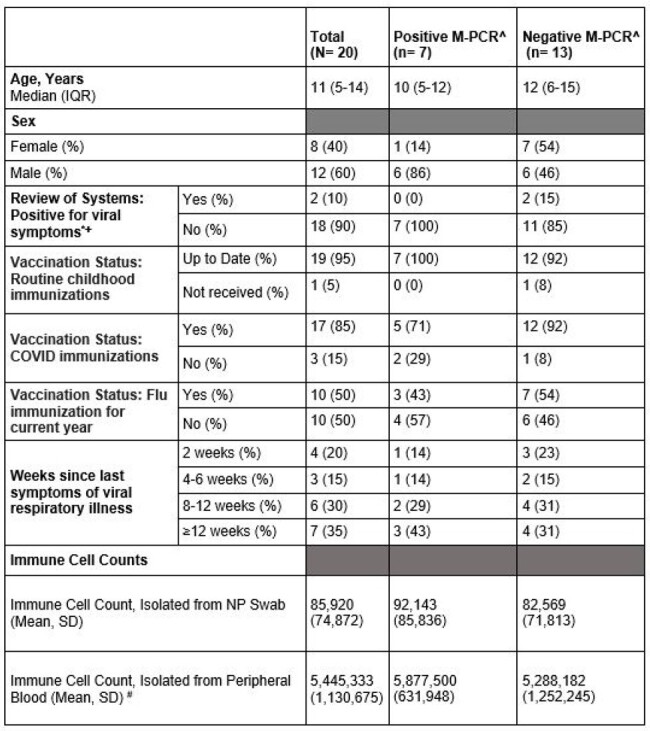

Legend:

^= Nasopharyngeal specimens were processed using the BioFire® Respiratory 2.1 (RP2.1) Panel

* = within 10 days of the participant’s enrollment visit

+ = Symptoms queried: fever, headache, runny or dripping nose, sore or painful throat, trouble breathing/shortness of breath, chest tightness/pain, dizziness, coughing, nausea, vomiting, diarrhea, clingy, fussy, decreased energy level

#= Among 15 participants where blood sample was available; 4 of 7 participants with positive M-PCR and 11 of 13 participants with negative M-PCR

NP=Nasopharyngeal; SD= Standard deviation; M- PCR= Multiplex Polymerase Chain Reaction

**Methods:**

The ongoing Pediatric Respiratory Co-infection and Immunologic Response Study (Peds RECON) measures clinical and immune responses during respiratory viral infections in participants aged 1 month to 17 years, enrolled while healthy, with follow-up visits during illness over two years. Enrollment involves demographic data collection, a 10-day respiratory symptom review, and sample collection (nasopharyngeal (NP) swab and peripheral blood (PB)). NP swabs undergo M-PCR using the BioFire® Respiratory 2.1 panel, and both NP swabs and PB samples undergo immune cell quantification. A prevalence rate was calculated based on M-PCR-positive NP swabs per all collected NP swabs. A t-test compared NP immune cell counts between participants with and without RVPD at enrollment, while logistic regression assessed the association with RVS and RVPD.

Participant Findings by Multiplex PCR Positivity in 20 Peds RECON Participants at Enrollment

**Figure:**
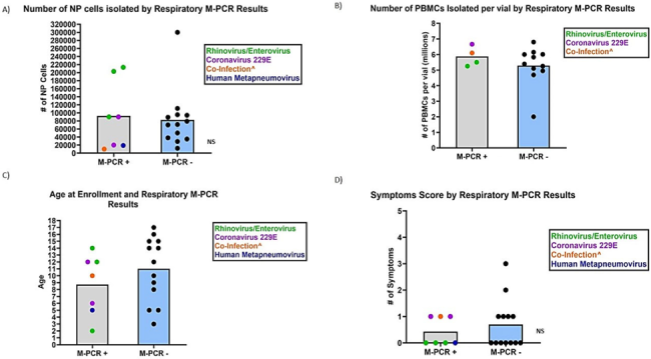

Legend:

+= positive

-= Negative

^= Rhinovirus/Enterovirus and Parainfluenza 3

#=Number

NS= not significant, p-value ≥ 0.05

Peds RECON- Pediatric Respiratory Co-infection and Immunologic Response Study

NP= Nasopharyngeal (NP)

M-PCR= Multiplex Polymerase Chain Reaction, Nasopharyngeal specimens were processed using the BioFire® Respiratory 2.1 (RP2.1) Panel

PBMC=Peripheral Blood Mononuclear Cells

**Results:**

From January to May 2024, 20 children (median age: 11 years) were enrolled (table). Most reported respiratory illness ≥4 weeks prior (n=16). Seven NP swabs tested positive among 20 NP swabs (35% prevalence rate), primarily rhinovirus/enterovirus. At enrollment, 45% reported at least one respiratory symptom in the preceding 10 days. The mean nasal immune cell count was 85,920 (SD: 74,872), with no significant association between RVPD and immune cell counts (t (18) =0.27, p=0.80) or RVS (OR: 0.88; 95% CI: 0.14; 5.58) (figure A; D).

**Conclusion:**

Thirty-five percent of apparently healthy children in Peds RECON had a RVP detected on M-PCR, highlighting the discrepancy between clinical appearance and RVPD. Nasopharyngeal cell isolation did not correlate with positive M-PCR or reported symptoms, demonstrating M-PCR positivity for certain pathogens is indicative of asymptomatic presence of viral RNA. Further research is required to determine if asymptomatic shedding occurs or if the sensitivity of the assay for certain pathogens requires optimization.

**Disclosures:**

All Authors: No reported disclosures

